# Delayed Traumatic Diaphragmatic Hernias: Tomorrow’s Emergency

**DOI:** 10.7759/cureus.91424

**Published:** 2025-09-01

**Authors:** Gerard V Giangrosso, Thomas Cassier, Yuki Nakao, David Denning, Andrew J Weaver

**Affiliations:** 1 General Surgery, Marshall University Joan C. Edwards School of Medicine, Huntington, USA; 2 Trauma and Surgical Critical Care, Marshall University Joan C. Edwards School of Medicine, Huntington, USA

**Keywords:** acute care surgery and trauma, delayed traumatic diaphragmatic hernia, multiple rib fractures, post-trauma surveillance, traumatic diaphragmatic hernia

## Abstract

Traumatic diaphragmatic injury (TDI) is an often-overlooked consequence of blunt thoracoabdominal trauma. Despite advances in imaging, many diaphragmatic injuries are not identified on initial trauma CTs, leading to delays in diagnosis and possibly life-threatening complications. While some cases present acutely with respiratory distress, others remain undetected for years, progressing to delayed traumatic diaphragmatic hernia (dTDH). In this review, we discuss TDI and present two cases of delayed hernias that presented to the hospital and required urgent surgical intervention. However, as opposed to immediate presentation or long delayed presentation, these hernias developed in the intermediate timeframe. As such, we discuss the need for a structured follow-up for those at high risk of a traumatic hernia.

## Introduction

A missed injury today can become a life-threatening emergency tomorrow. Traumatic diaphragmatic injury (TDI) is an often-overlooked consequence of blunt thoracoabdominal trauma, occurring in approximately 5% of cases [1]. Despite advances in imaging, as many as 66% of TDIs are not identified on initial CT scans, leading to delays in diagnosis and, in some instances, life-threatening complications [2]. While some cases present acutely with respiratory distress, others remain undetected for months or even years, progressing to delayed traumatic diaphragmatic hernia (dTDH) [3]. Once herniation occurs, abdominal organs can become strangulated, leading to ischemia, necrosis, sepsis, and respiratory failure. These conditions demand urgent surgical intervention [4].

Although dTDH has been well-documented, its actual incidence remains difficult to quantify due to underreporting, particularly in cases where symptoms develop gradually [3]. Published studies report mortality rates for treated patients as high as 21% and 31% in untreated or complicated cases [5,6]. Known risk factors include lower rib fractures, blunt force trauma to the abdomen, and missed diaphragmatic injuries during the initial trauma assessment [1].

Most prior reports categorize dTDH into either acute or chronic presentations, often highlighting cases diagnosed at the time of initial trauma or several years later. The cases in this report illustrate a less frequently discussed timeline, one that bridges these two extremes. The first case was identified within days of injury, while the second developed gradually over five months despite prior rib fixation. These findings suggest that even when the initial injury appears to be stabilized, the risk of delayed herniation remains, underscoring the need for ongoing clinical vigilance and follow-up in trauma patients.

## Case presentation

TJ was a 73-year-old female who initially presented to an outside hospital after a syncopal fall from standing height. She received pan trauma scans, which demonstrated a left-sided moderately displaced 8-10 rib fracture and a trace left hemopneumothorax. No obvious diaphragmatic hernia or elevated hemi-diaphragm was visualized. The patient was then transferred to our facility for further trauma management. She was initially treated with pulmonary toilet and multi-modal pain control, and no chest tube was needed. No repeat imaging was performed afterwards. She progressed well with relatively minimal pain and was discharged on hospital day two.

**Figure 1:**
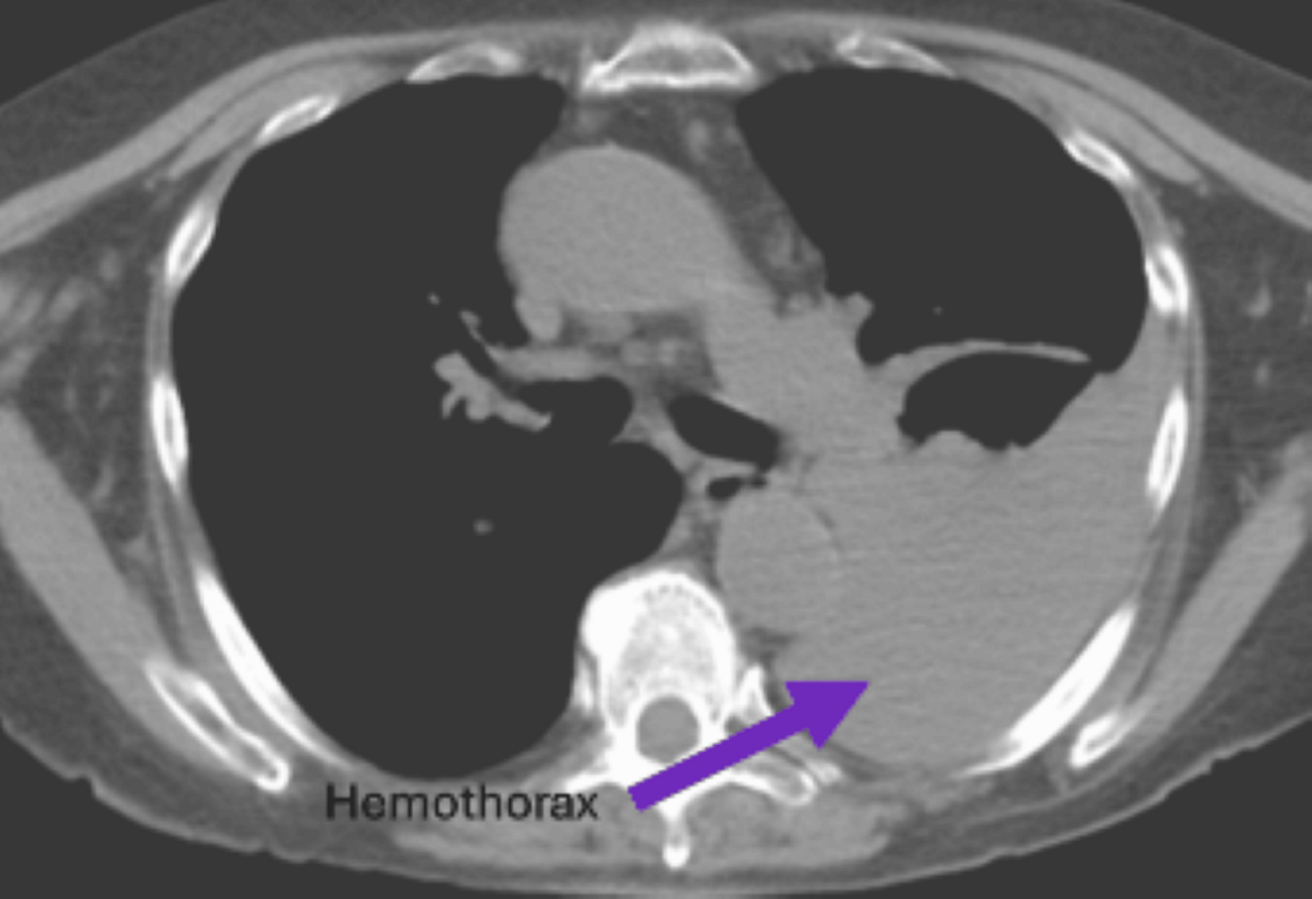
Left-sided hemothorax with possible hiatal hernia.

The patient re-presented to the outside hospital emergency department one week later with increased shortness of breath and chest pain. A new CT scan demonstrated a moderate left-sided fluid collection concerning for retained hemothorax and a possible left-sided diaphragmatic hernia (Figure 1). She was transferred to our facility for repeat evaluation. Given her shortness of breath with a moderate-sized hemothorax, a chest tube was placed, which returned 900 cc of old blood. The patient was then scheduled for a video-assisted thoracoscopic surgery (VATS), hemothorax washout, possible rib plating, and possible repair of a diaphragmatic hernia. Intraoperatively, a 4 cm diaphragmatic hernia with incarcerated omentum was noted, but there were no signs of strangulation. The omentum was reduced, and the hernia was closed primarily using 0-Ethibond suture. No mesh was used. She progressed well postoperatively and was eventually discharged to a rehab facility on postoperative day eight. She was seen in the clinic two weeks after discharge with no issues.

CH was a 69-year-old female who was transferred to our institution after a motor vehicle collision. She was restrained, but the speed of the vehicle was unknown. The patient was found to have a left-sided pneumothorax, as well as left two through nine rib fractures visualized on CT scan. A chest tube was placed, which improved the pneumothorax. The patient was then admitted to the ICU for pain control and pulmonary toilet. While in the ICU, her pain was semi-controlled with poor inspiratory effort. Two days later, the decision was made to perform operative fixation of her rib fractures, and she subsequently underwent an extra-thoracic approach reduction and fixation of ribs three through eight. The intercostal muscles were noted to be severely damaged due to her fractures, and because of this, a view into the thorax was easily seen with no obvious intra-abdominal organs visualized. The diaphragm was easily visualized with no defect. The patient’s pain and respiratory effort improved, and she was subsequently transferred to the floor and discharged.

**Figure 2:**
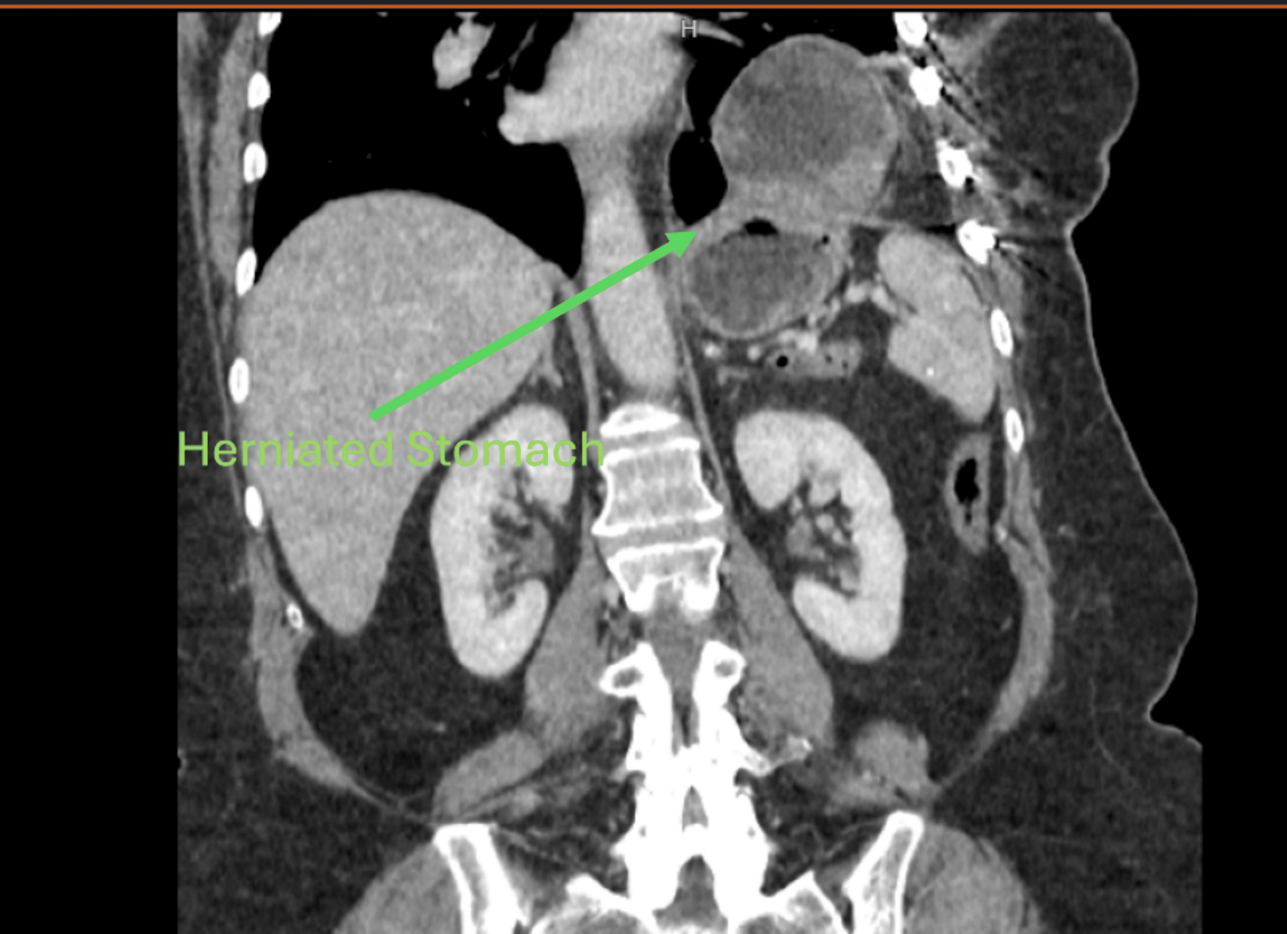
Left-sided diaphragmatic hernia.

Five months later, the patient presented to the emergency department (ED) with approximately one day of nausea/vomiting and left upper quadrant abdominal pain. A CT of the abdomen and pelvis was performed by ED providers, which visualized a left-sided diaphragmatic hernia with herniation of the stomach into the chest (Figure 2). The patient was brought to the operating theater for a VATS with traumatic diaphragmatic hernia repair. Dense thoracic adhesions were lysed, and the stomach was unable to be fully reduced into the chest. As a result, conversion to open thoracotomy was performed. The hernia defect was then enlarged by 1 cm, and then the stomach was reduced into the abdomen. After reduction, evaluation of the hernia was possible. The defect was in the middle of the left dome of the diaphragm, approximately 6 cm in length. Most likely, the initial injury was a very small tear, but progressive activity of the diaphragm led to this defect becoming larger over the months. The diaphragm was then repaired primarily with 0-Prolene using horizontal mattress sutures. A chest tube was placed, and then the patient was extubated and brought to the post-anesthesia care unit (PACU) in stable condition. The chest tube was removed on postoperative day five after chest tube output was appropriate. She was then discharged on postoperative day six. The patient was seen in the clinic one month later and had no complaints.

## Discussion

Delayed traumatic diaphragmatic hernia (dTDH) remains a challenging diagnosis that often goes undetected at the time of initial trauma evaluation. Mechanistically, TDI results from a sudden surge in intra-abdominal pressure that tears the diaphragm, most commonly at its posterolateral aspect, where the muscle is structurally weakest [1]. Many of these defects are not apparent at first, as the injury may be concealed by adjacent organs or a temporary sealing effect. Over time, negative intrathoracic pressure and gradual shifts in abdominal contents contribute to the herniation process, which may take days, months, or even years to manifest [2,7].

The difficulty in diagnosing TDI early is challenging for clinicians. Despite improvements in multidetector computed tomography (MDCT), studies show that more than half of cases are overlooked on initial imaging [2]. This can be attributed to factors such as small, self-contained tears, hemothorax or pneumothorax obscuring visualization, and the delayed onset of herniation [3]. Consequently, trauma patients with lower rib fractures, a known risk factor for diaphragmatic injury, should be monitored closely, even if their initial imaging appears unremarkable [1]. When these patients later develop unexplained respiratory or gastrointestinal symptoms, a repeat imaging evaluation should be considered to rule out evolving dTDH. Diagnostic laparoscopy is an option for evaluation, but surgeons may be opposed to performing surgery unless there is clear evidence for an injury.

The cases presented here reveal an underrecognized pattern of dTDH progression. In the first case, early reassessment of respiratory symptoms enabled timely detection and successful minimally invasive repair. The second case, however, developed over five months, despite prior rib fixation, and ultimately required conversion from thoracoscopic to open thoracotomy due to an incarcerated stomach. These cases suggest that dTDH is not strictly confined to either acute or long-delayed presentations, but rather, can develop gradually within a mid-range timeframe that may not be captured by traditional follow-up practices.

Most existing literature emphasizes dTDH occurring either immediately post trauma or years later when complications arise. However, our cases indicate that patients may remain at risk for herniation even after an initial period of stability. This raises the question of whether structured follow-up imaging at defined intervals, rather than symptom-driven reassessment, might allow for earlier identification and intervention before more complex surgical procedures are required.

Given the limitations of symptom-based monitoring, a structured follow-up protocol may be warranted for high-risk patients, particularly those with multiple rib fractures or those requiring surgical fixation of the ribs. Scheduled CT imaging at predefined intervals, such as four to six weeks post injury for two years, could improve early detection and facilitate less invasive interventions. However, we acknowledge that this would be difficult for hospitals and insurance companies to get on board with. Future research should evaluate the cost-effectiveness and clinical impact of routine follow-up imaging in preventing delayed surgical complications.

## Conclusions

Delayed traumatic diaphragmatic hernia is a serious consequence of missed diaphragmatic injuries. Some cases of diaphragmatic injuries could easily be missed on initial imaging and manifest later. The cases presented here illustrate that even in patients who initially appear without an obvious injury, dTDH can develop gradually. This challenges the conventional classification of dTDH as either acute or chronic and highlights the need for a consensus in regards to follow-up to ensure recognition of these delayed injuries.

Early recognition through proactive CT imaging strategies may allow for timely intervention before complications arise. Future efforts should focus on defining optimal follow-up protocols to improve detection and prevent the need for emergent surgical management. A more proactive approach to post-trauma surveillance is essential in preventing missed diagnoses from becoming critical emergencies.
